# Should Prediabetes Be Classified as a Treatable Disease?

**DOI:** 10.3390/jcm15020710

**Published:** 2026-01-15

**Authors:** William E. Winter, Ishwarlal Jialal

**Affiliations:** 1 Department of Pathology, Immunology and Laboratory Medicine, University of Florida, Gainesville, FL 32610, USA; winter@pathology.ufl.edu; 2 Department of Medicine and Pathology, UC Davis School of Medicine, 2616 Hepworth Drive, Davis, CA 95618, USA

**Keywords:** prediabetes, cardiovascular disease, intensive lifestyle changes, microvascular complications

## Abstract

Prediabetes is a serious and major global problem afflicting approximately 21% of the world’s population. It is the intermediate stage between normal glucose levels and type 2 diabetes mellitus (T2DM). Prediabetes is associated with major complications including the development of T2DM and increased cardiovascular disease (CVD). It can be easily diagnosed with an inexpensive plasma glucose level and/or a hemoglobin A1c (HbA1c) measurement. The mainstay of treatment is intensive lifestyle (ILS) intervention, including reduction in calories, especially saturated fats, refined carbohydrates, etc., coupled with regular physical activity of 150 min per week since ILS changes, with at least a 5% weight loss, have been shown to reduce progression to T2DM in multiple studies globally. Also, metformin therapy has been shown to prevent the progression to T2DM. In conclusion, serious consideration by guideline committees to classify prediabetes as a disease is highly recommended based on its global burden, easy and cost-effective diagnosis, association with serious conditions of diabetes and CVD, and effective ILS intervention. Therapy targeting those at an especially high risk for T2DM, such as persons with impaired glucose tolerance (IGT), impaired fasting glucose (IFG) with values ≥ 110 mg/dL (6.1 mmol/L), and/or HbA1c ≥ 6.0% (42 mmol/mol) coupled with overweightness or obesity.

## 1. Introduction

As adapted from Oxford Languages, human disease can be defined as “a disorder of structure or function, especially one that has a distinctive group of symptoms, signs, or anatomical changes, and often a known cause.” Based on the recognized relationship of prediabetes to subsequent macrovascular, microvascular and neuropathic complications, prediabetes should be classified as a “disease.” However, this is not without controversy.

Prediabetes is a major and growing global problem due to a sedentary lifestyle and consumption of a high-calorie, unhealthy diet, overweightness, aging of the population, family histories of diabetes, poor sleep quality, stresses of everyday life, and various genetic factors [1,2,3]. The International Diabetes Federation (IDF) estimated in 2024 that 635 million adults had impaired glucose tolerance (IGT) and an additional 488 million adults had impaired fasting glucose (IFG) [1]. Therefore, the diagnosis, treatment, and prevention of prediabetes warrant the increased attention of both patients and health professionals. Furthermore, treatment of prediabetes prevents T2DM which is a major global epidemic causing adverse medical, sociological, and economic consequences.

## 2. Risk Factors

Risk factors for disease can be described as “continuous” or “discontinuous.” For example, regarding smoking and the development of squamous cell carcinoma of the lung, the risk of this cancer is 13-fold higher in smokers than in nonsmokers. Therefore, smoking serves as a discontinuous risk factor sorting risk for squamous cell carcinoma of the lung into two distinctly different groups: a high-risk group (smokers) and a low-risk group (nonsmokers).

In contrast, especially concerning vascular diseases, risk factors for adverse outcomes such as myocardial infarction or stroke are continuous variables: with increasing plasma glucose (PG), blood pressure, and low-density-lipoprotein (LDL) cholesterol, there is a continuously increasing risk of adverse vascular outcomes. Concerning PG, it is then challenging to assign the most clinically appropriate PG or hemoglobin A1c (HbA1c) cut points that define “prediabetes” as a disease. Risk factors for prediabetes are similar to the risk factors for T2DM. Such risk factors include excess body weight, age 45 years and greater, ethno-genetic predilection, socio-economic disadvantage, inactivity, harmful diet, fatty liver, hypertension, a history of gestational diabetes in women, and certain medications such as thiazide diuretics, second-generation antipsychotic medications, certain HIV medications, etc. [1,2,3,4,5].

The current cut points defining diabetes using fasting plasma glucose (FPG), 2 h PG (2hPG) concentrations on 75 g oral glucose tolerance testing (OGTT), and HbA1c levels were derived, in part, from outcome studies in Pima Native Americans, Egyptians, and 40-to-74-year-old NHANES III participants. In each population, an inflection point of significantly increased risk of retinopathy was observed that defined the 2hPG cutoff for hyperglycemia as ≥200 mg/dL (11.1 mmol/L), and a HbA1c cutoff for hyperglycemia of ≥6.5% (48 mmol/mol) [1,3]. Until 1997, the American Diabetes Association (ADA) had defined fasting hyperglycemia as a fasting PG (FPG) of ≥140 mg/dL (7.8 mmol/L). However, in 1997, the ADA lowered the PG cutoff for hyperglycemia to ≥126 mg/dL (7.0 mmol/L) to more closely correlate FPG-defined hyperglycemia with hyperglycemic defined as a 2hPG of ≥ 200 mg/dL (11.1 mmol/L) [1,3].

## 3. Definition of Prediabetes

**Figure 1:**
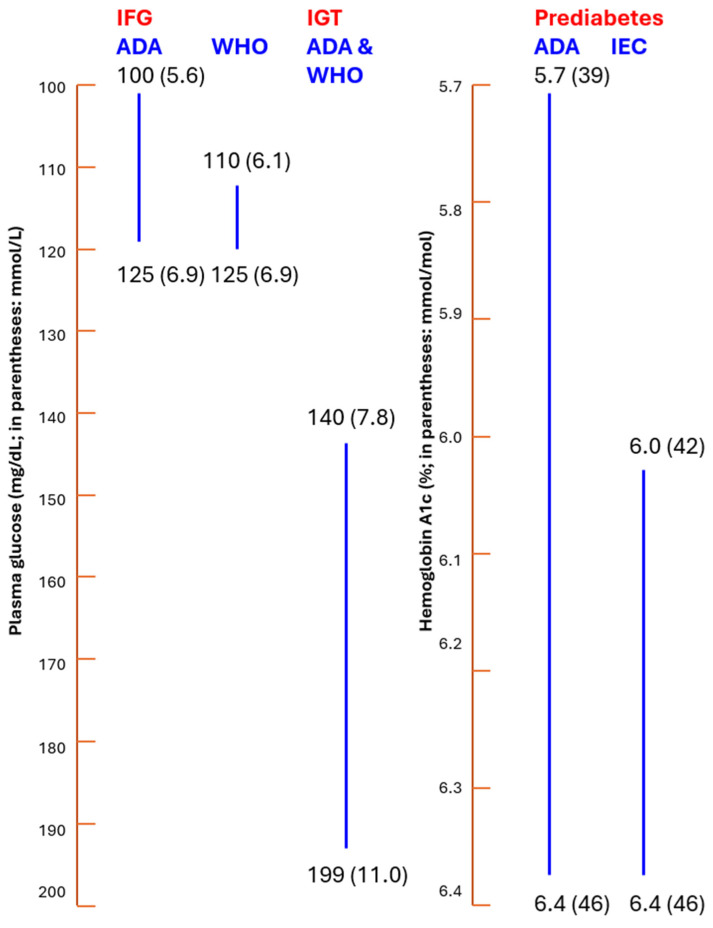
ADA, WHO, and IEC criteria.

If prediabetes is indeed a disease, what glycemic cut points should then be used to diagnose prediabetes? The 2025 definitions of prediabetes offered by the ADA, the World Health Organization (WHO), and the International Expert Committee (IEC) lack consensus, as depicted in Figure 1 [1,3,4]. Thus, the classification of prediabetes is controversial hindering its classification as a disease entity. Whilst both the ADA and WHO are in agreement for the 2hPG following an OGTT (140–199 mg/dL, 7.8–11.0 mmol/L), they differ with respect to the lower PG levels for IFG; the ADA criteria is 100–125 mg/dL (5.6–6.9 mmol/L) and the WHO criteria is 110–125 mg/dL (6.1–6.9 mmol/L). Also, the ADA uses HbA1c as an additional criterion with levels of 5.7–6.4% (39–46 mmol/mol). However, the WHO does not use HbA1c as a criterion. In contrast, the International Expert Committee (IEC) provides only prediabetes cut points for HbA1c of 6.0–6.4% (42–46 mmol/mol) [1,3,4,5].

These criteria are summarized in Figure 1.

Using the ADA definitions of prediabetes [3], several permutations of abnormalities can be defined regarding IFG (FPG: 110–125 mg/dL; 6.1–6.9 mmol/L) and IGT (2hPG: 140–199 mg/dL; 7.8–11.0 mmol/L). Because HbA1c is a measure of long-term glycemia, and because an elevated HbA1c (e.g., ≥6.5%; 48 mmol/mol) is not an earlier marker of hyperglycemia than an elevated FPG or 2hPG, HbA1c is not usually included in such “combined” definitions of prediabetes and furthermore the assay has certain limitations [1,3,4,5].

In a 2024 International Diabetes Federation (IDF) position paper, a data-based expert committee proposed the use of a 1 h level of ≥155 mg/dL (8.6 mmol/L) on an OGTT as the best predictor of diabetes because this concentration identified otherwise normoglycemic people at the highest likelihood of progressing to diabetes [6]. However, moving such testing into clinical practice would require a major revision of the OGTT necessitating the addition of a 1 h draw time to the now standard 2 h OGTT, or replacement, altogether, of the 2 h OGTT by a 1 h OGTT. Thus, much further study and discussion would be required prior to global adoption of this suggested IDF recommendation.

Because vascular disease risk is continuous, the authors believe that it might be most advantageous to define prediabetes with the broadest set of cut points as proposed by the ADA [3]. This would provide greater sensitivity for the detection of prediabetes. Nevertheless, each unique cut point (IFG versus IGT) does not identify identical populations [4,5], and different populations are also identified using different organizational cut points (ADA, WHO, and/or IEC).

One might then inquire, why are not the same populations of persons identified by the FPG and 2hPG cut points? The answer to this question is that the pathophysiology of prediabetes is heterogeneous [2]. Hepatic insulin resistance and normal muscle insulin sensitivity coupled with impairment of first phase insulin secretion are most commonly observed in persons with isolated IFG. In contrast, moderate to severe skeletal muscle insulin resistance coupled with decreases in both first and second phase insulin secretion, with hepatic insulin resistance being normal to slightly reduced, characterizes persons with isolated IGT [1,3,4,5].

## 4. Complications of Prediabetes

Prediabetes is an important disorder because it is associated with multiple serious complications, as summarized in Table 1. The best-known complication of prediabetes that has been established in populations globally is the increased risk of developing T2DM [1,3,4]. In the U.S., approximately 10% of persons with prediabetes yearly progress to T2DM [4]. Nevertheless, not everybody with prediabetes progresses to T2DM [1]. As detailed above, there are certain risk factors that increase the propensity to T2DM.

Individuals with prediabetes have more cardiovascular (CV) risk factors such as obesity, atherogenic dyslipidemia (comprising an increase in triglycerides, non-HDL-cholesterol, apolipoprotein B levels, and a preponderance of small dense LDL particles) [7], and hypertension because there is great overlap with the metabolic syndrome [1]. A meta-analysis of prospective studies with a median follow-up of 9.8 years demonstrated an increased rate of CVD in persons with prediabetes. CVD includes coronary artery disease, stroke, and peripheral arterial disease [4,5]. Mechanisms that predispose individuals to the increased risk of CVD include increases in insulin resistance, inflammation, oxidative stress, endothelial dysfunction, and a pro-thrombotic state [7].

Prediabetes is a risk factor for the growing epidemic of metabolic dysfunction-associated steatotic liver disease (MASLD) [8], which further exacerbates the pro-inflammatory state and insulin resistance predisposing persons to prediabetes [1,8]. Numerous studies support an increased risk for microvascular complications, including diabetic nephropathy, retinopathy, and neuropathy in prediabetes [1,2,3,4,5]. Furthermore, prediabetes has been associated with impaired cognition [1,3,4]. In a recent important report by Rooney et al. [9], in over 10,000 participants in the Atherosclerosis Risk in Communities (ARIC) study, with a 30-year follow-up, they showed an increased risk for CVD, heart failure, chronic kidney disease (CKD), and mortality with prediabetes even after accounting for progression to T2DM.

## 5. Treatment

There is abundant data demonstrating that therapy of prediabetes can delay progression to T2DM. The cornerstone of therapy for prediabetes is intensive lifestyle (ILS) changes focusing on a reduction in unhealthy calories such as saturated fats, refined carbohydrates, etc., coupled with moderate exercise of 150 min per week resulting in weight loss of at least 5% [1,3].

Numerous studies from various parts of the world including the U.S.A., China, Finland, India, etc., showed that weight loss with lifestyle changes delayed progression to diabetes [1,2,3,4,10,11,12]. The largest study (n = 3234 adults) was from the U.S., the Diabetes Prevention Program (DPP), which showed that weight loss of 7% with ILS changes resulted in a 58% reduction in the incidence of diabetes over a mean duration of 2.8 years in persons with a BMI of ≥24 kg/m^2^ [10,12]. Recently, both Knowler and Crandall summarized the durability of this benefit over the 21-year extension of the DPP study, DPP-Outcomes Study (DPPOS) [10,11]. The benefit of reduced diabetes progression compared to the placebo group was 24%. During this long-term follow-up there was no benefit in microvascular disease, CV outcomes, cancer, or cognitive scores. However, compared to those that progressed to T2DM, those who did not progress had a significant 28% reduced prevalence of microvascular diseases [11]. It should be pointed out that the Da Qing Study from China reported a 39% reduction in progression to T2DM over 30 years [12], and the Finnish study reported a 43% reduction in the development of T2DM over 7 years [12]. All the above studies with duration spanning 7–30 years underscore the durability of the benefit of ILS changes in preventing T2DM progression. The Da Qing study also reported a 33% decrease in CV mortality and 35% lower risk of microvascular complications in the ILS intervention group [12]. In a recent report, Vazquez Arreola et al. [13] using both the DPPOS and Da Qing cohorts tested if remission to normoglycemia had an effect on CV death and hospitalization for heart failure. They showed in each cohort separately and in the combined cohort significantly reduced risk of CV death or hospitalization for heart failure. They also showed a reduction of visceral fat coupled with an amelioration in both insulin resistance and low-grade inflammation. Sadly, only a minority of patients with prediabetes achieved remission in both studies (11.4 and 13.3%, respectively). They requested that future studies give priority to the majority of patients who did not achieve remission possibly integrating ILS and cost-effective pharmacotherapy.

Another cost-effective strategy is the use of the drug metformin, which is generic and globally available. In the DPP and DPPOS metformin constituted one study arm and reduced progression to T2DM by 31% in the DPP study at 2.8 years and produced a 17% reduction at 21 years in DPPOS [10,11,12]. However, metformin had no benefit on microvascular or CV diseases, or cognitive scores. Also, metformin appeared to be most beneficial in persons < 60 years of age such that the ADA guidelines recommend its use in this age group with BMI ≥ 35 kg/m^2^, plasma glucose ≥ 110 mg/dL (6.1 mmol/L), and HbA1c ≥ 6.0% (42 mmol/mol) and in individuals with prior gestational diabetes [12]. Both of the above strategies of ILS intervention and metformin are cost-effective and provide cost savings compared to the placebo [12].

Numerous other pharmacotherapies have been shown to prevent progression to T2DM including alpha-glucosidase inhibitors, thiazolidinediones, vitamin D, glucagon-like peptide 1 (GLP-1) receptor agonists, and combined GLP-1 and glucose-dependent insulinotropic peptide (GIP) receptor agonists [1,3,5]. Vitamin D supplementation appears to be a reasonable strategy in some studies [1]. Pioglitazone for patients with prediabetes and MASLD could also prove beneficial in mitigating the progression of both disorders provided that there are no contra-indications.

Finally, a very attractive and alluring treatment is the ushering in of the GLP-1 and GLP-1/GIP agonists for persons with prediabetes and obesity. However, the present prohibitive costs reduce their appeal and cost-effectiveness globally despite their great benefit for weight loss and in providing CVD protection [1,4,5,11].

## 6. Conclusions

In conclusion, serious consideration needs to be given to elevating prediabetes, a mammoth global problem, to a disease state based on the collective data of its associated severe complications, ease of diagnosis which is inexpensive, and effective therapies (especially ILS changes) that are available that can forestall the progression to T2DM and the sequela of T2DM. Since an important percentage of patients with prediabetes revert to normoglycemia [1,2] therapy should be targeted to those at highest risk for T2DM such as individuals with IGT, IFG with values ≥110 mg/dL (6.1 mmol/L), and those with HbA1c ≥ 6.0% (42 mmol/mol), especially if they are overweight/obese. There is an urgent need for improved biomarkers or biomarker panels based on OMICS and microRNAs to better identify persons with prediabetes that progress to T2DM and/or manifest CVD.

Also introducing moderate dose statin therapy in addition to ILS changes to maintain the LDL-C <100 mg/dL (2.6 mmol/L) and adding ACE-inhibitor/ARB therapy to ILS changes to maintain blood pressure < 130/80 mmHg will potentially mitigate the future risk for CV diseases which would be an important departure from the largely gluco-centric focus to date. A crucial study in this population is testing the effect of combining a statin with an ACE-inhibitor/ARB on the reduction in CVD events since both are potent risk factors for CVD.

## Figures and Tables

**Table 1:** Complications associated with prediabetes.

Diabetes
Cardiovascular disease
Microvascular disease, e.g., Nephropathy
Metabolic dysfunction-associated liver disease (MASLD)-cirrhosis
Cognitive decline
Cancers associated with obesity

## Data Availability

The data is available from the senior author for review on reasonable request.

## References

[1] Unnikrishnan R., Shaw J.E., Chan J.C.N., Wild S.H., Peters A.L., Orrange S., Roden M., Mohan V. (2025). Prediabetes. Nat. Rev. Dis. Primers.

[2] The Lancet Diabetes Endocrinology (2025). Prediabetes: Much more than just a risk factor. Lancet Diabetes Endocrinol..

[3] American Diabetes Association Professional Practice Committee for Diabetes (2026). Diagnosis and Classification of Diabetes: Standards of Care in Diabetes—2026. Diabetes Care.

[4] Echouffo-Tcheugui J.B., Perreault L., Ji L., Dagogo-Jack S. (2023). Diagnosis and Management of Prediabetes: A Review. JAMA.

[5] Olatunbosun S.T., Winter W.E. (2025). A Review of Prediabetes: Diagnosis, Consequences and Interventions. Int. J. Clin. Transl. Med..

[6] Bergman M., Manco M., Satman I., Chan J., Schmidt M.I., Sesti G., Vanessa Fiorentino T., Abdul-Ghani M., Jagannathan R., Kumar Thyparambil Aravindakshan P. (2024). International Diabetes Federation Position Statement on the 1-hour post-load plasma glucose for the diagnosis of intermediate hyperglycaemia and type 2 diabetes. Diabetes Res. Clin. Pract..

[7] Chait A., Eckel R.H., Vrablik M., Zambon A. (2024). Lipid-lowering in diabetes: An update. Atherosclerosis.

[8] Tilg H., Petta S., Stefan N., Targher G. (2026). Metabolic Dysfunction-Associated Steatotic Liver Disease in Adults: A Review. JAMA.

[9] Rooney M.R., Wallace A.S., Tcheugui J.B.E., Fang M., Hu J., Lutsey P.L., Grams M.E., Coresh J., Selvin E. (2025). Prediabetes is associated with elevated risk of clinical outcomes even without progression to diabetes. Diabetologia.

[10] Knowler W.C., Doherty L., Edelstein S.L., Bennett P.H., Dabelea D., Hoskin M., Kahn S.E., Kalyani R.R., Kim C., Pi-Sunyer F.X. (2025). Long-term effects and effect heterogeneity of lifestyle and metformin interventions on type 2 diabetes incidence over 21 years in the US Diabetes Prevention Program randomised clinical trial. Lancet Diabetes Endocrinol..

[11] Crandall J.P., Dabelea D., Knowler W.C., Nathan D.M., Temprosa M., DPP Research Group (2025). The Diabetes Prevention Program and Its Outcomes Study: NIDDK’s Journey into the Prevention of Type 2 Diabetes and Its Public Health Impact. Diabetes Care.

[12] American Diabetes Association Professional Practice Committee for Diabetes (2026). Prevention or Delay of Diabetes and Associated Comorbidities: Standards of Care in Diabetes—2026. Diabetes Care.

[13] Vazquez Arreola E., Gong Q., Hanson R.L., Wang J., Sandforth L., He S., Sandforth A., Qian X., Giacca M., Bornstein S.R. (2026). Prediabetes remission and cardiovascular morbidity and mortality: Post-hoc analyses from the Diabetes Prevention Program Outcome study and the DaQing Diabetes Prevention Outcome study. Lancet Diabetes Endocrinol..

